# A Role of Glucose Overload in Diabetic Cardiomyopathy in Nonhuman Primates

**DOI:** 10.1155/2021/9676754

**Published:** 2021-03-30

**Authors:** Xiu Wang, Shi Jin, Weina Hu

**Affiliations:** ^1^Department of Anesthesiology, The Fourth Affiliated Hospital of China Medical University, Shenyang 110034, China; ^2^Department of Endocrinology, The Fourth Affiliated Hospital of China Medical University, Shenyang 110034, China; ^3^Department of Cardiology, The Fourth Affiliated Hospital of China Medical University, Shenyang 110034, China

## Abstract

Type 2 diabetes (T2D) plays a major role in the development of heart failure. Patients with T2D have an increased risk to develop HF than healthy subjects, and they always have very poor outcomes and survival rates. However, the underlying mechanisms for this are still unclear. To help develop new therapeutic interventions, well-characterized animal models for preclinical and translational investigations in T2D and HF are urgently needed. Although studies in rodents are more often used, the research findings in rodents have often failed to be translated into humans due to the significant metabolic differences between rodents and humans. Nonhuman primates (NHPs) serve as valuable translational models between basic studies in rodent models and clinical studies in humans. NHPs can recapitulate the natural progress of these diseases in humans and study the underlying mechanism due to their genetic similarity and comparable spontaneous T2D rates to humans. In this review, we discuss the importance of using NHPs models in understanding diabetic cardiomyopathy (DCM) in humans with aspects of correlations between hyperglycemia and cardiac dysfunction progression, glucose overload, and altered glucose metabolism promoting cardiac oxidative stress and mitochondria dysfunction, glucose, and its effect on cardiac resynchronization therapy with defibrillator (CRT-d), the currently available diabetic NHPs models and the limitations involved in the use of NHP models.

## 1. Introduction

The prevalence of worldwide type 2 diabetes (T2D) is increasing exponentially across age and gender. It is estimated that the number of global T2D patients has reached 425 million in 2017 [1]. T2D, one of the main cardiovascular (CV) risk factors, contributes to the yearly increased CV morbidity and mortality [2, 3]. Heart failure and T2D are always accompanied by each other in clinical practice. Around 20%-40% of HF patients are having T2D [4, 5]. Patients with T2D have more than twice folds of risk developing HF than people without diabetes [6–9]. The increased prevalence of HF and the poor outcomes and survival rate of HF patients make it the most worrying diabetes complications over several decades [10]. Diabetic cardiomyopathy (DCM) is now recognized as a separate myocardial disease manifested as impaired cardiac functions independent of coronary artery disease, atherosclerosis, and hypertension [11–13]. Cardiac magnetic resonance imaging (cardiac MRI or CMR) [14] and echocardiography [15] have been used as the gold standards to evaluate the cardiac structure, ventricular function, and tissue characterization noninvasively. There are 2 distinct phenotypes of DCM: one is heart failure with preserved left ventricular (LV) ejection fraction (HFpEF), or diastolic dysfunction, often characterized by a normal ejection fraction (EF), abnormal ratio of the peak velocities at early (E), and late (A) diastole (E/A), a slow LV relaxation, thick LV walls, and elevated LV filling pressures [13]. This phenotype may precede the development of systolic dysfunction [16, 17], named heart failure with reduced left ventricular ejection fraction (HFrEF), which is characterized as a reduced EF, enlarged LV cavity, and impaired contractility. T2D is closely associated with those 2 phenotypes of cardiomyopathy in humans [18]. Recent studies have shown that poor glycemic control correlates with increased risk of HF in diabetic patients, and the myocardial changes in those patients are believed to be induced by hyperglycemia [19, 20]. T2D was known to be one of the main risk factors and determinants of HFrEF. A recent study has reported the detrimental epigenetic effects of T2D on the cardiac pump and cardiac remodeling process in HF patients. MicroRNAs (miRs), a family of small regulatory noncoding RNAs, are implicated in epigenetic regulation of different aspects of cardiac structure and function such as cardiac fibrosis, apoptosis, and hypertrophy. Therefore, miRs were served as biomarkers for patients with both HF and T2D [21, 22]. T2D is also a heterogeneous disease, and the association between T2D and type 2 ryanodine receptor/Ca^2+^ release channel (RyR2), a key regulator for excitation-contraction coupling in the heart, has been identified by GWAS. It was reported that mutant leaky RyR2 channels were found in HF patients, and leaky RyR2 played a critical role in the pathogenesis of cardiac arrhythmias and impaired glucose metabolism [23]. Although significant progress has been achieved, the precise mechanism of DCM remains unclear. The foundation of these researches has been the use of small animals to uncover mechanisms of DCM and develop novel treatments. This review briefly discussed the common NHP models of T2D and HF and summarized the contributions of our understanding of glucose overload in DCM and drug discovery.

## 2. Finding an Ideal Animal Model

Ideally, the human-relevant T2D and HF research would be performed in humans but this is impossible due to technical and ethical considerations. Therefore, it is necessary to have an ideal model to recapitulate the human T2D and HF process in nonhuman systems. Although some of this can be done without the use of animals but through computer models or in vitro systems, these systems are incapable of reproducing the complex and multifactorial in vivo pathophysiology of T2D and HF.

Rodents are the most widely used animal species for various reasons. Practically, they are inexpensive, small in size, easy to breed, and genetically modifiable and have a short generation time and an accelerated lifespan. Furthermore, rodents are biologically similar to humans in many diseases and conditions and are well-established experimental models for studying the pathogenesis of diabetes-related cardiac dysfunction. However, several crucial differences between humans and rodents limit their potential as an ideal model [24, 25]. Among these disadvantages are the fact that rodents are only distantly related to humans, they do not naturally develop T2D, and are also resistant to diet-induced obesity, T2D, and cardiac dysfunction [26]. Rodent models have small size limitations, nocturnal activities, and they do not have menstrual cycles. These issues ultimately led to the use of animal models that more closely mimic humans.

## 3. Studies in Cardiac Mediated Cellular Pathways in Diabetes Nonhuman Primates (NHPs)

Although rodent models exhibit distinct advantages [27, 28], essential differences in the cardiometabolic process between rodents and humans have impeded direct translation of discoveries in rodents to humans. NHPs served as an ideal translational bridge between basic research and clinical application due to the findings from NHP studies are highly translatable to humans. Spontaneously, T2D and DCM develop in nonhuman primates (NHPs) just as it does in humans. NHPs exhibit clinical features of obesity, insulin resistance, diabetes, cardiac dysfunction, and pancreatic pathology that are observed the same in humans [29–33]. The cytoarchitecture and function of NHPs pancreatic islets that produce insulin are very similar to humans [34], making NHPs a critical model for T2D and DCM. Cardiac MRI and echocardiography have also been used to characterize cardiac functions in NHPs with T2D and DCM [31, 35, 36]. NHPs and humans have very similar systems in regulating blood glucose. Some researchers have shown that the incidence of low EF and abnormal E/A ratio in hyperglycemic NHPs was significantly higher than those in normal glycemic ones [31]. Consistently, there were also significant differences in the incidence of LV dysfunction between normal and high glycated hemoglobin (HbA1C) in NHPs [31]. Besides, NHPs have patterns of comorbidity that mirror humans and also exhibit long average life spans [37]. NHP studies also allow for complete control of the experimental environment including housing, experimental diet, and social interactions [28]. Furthermore, the use of NHPs studies meets the U.S. Food and Drug Administration (FDA) drug development requirement that toxicology testing should be conducted in a pharmacologically relevant species. Therefore, the NHP models are believed to be greatly suitable for preclinical drug development studies.

Cardiac metabolism and metabolic flexibility are impaired in the diabetic heart as evidenced by impaired glucose utilization and fatty acid oxidation both in humans and NHPs [33, 38]. This alteration leads to glucotoxicity and lipotoxicity, thus, further contribute to cellular dysfunction in all forms of obesity and diabetes, progression to DCM [39–41]. What is more, diabetes and dyslipidemia commonly occur together, with lipid abnormalities affecting two-thirds of T2D patients. High glucose accelerates atherosclerosis formation in the setting of diabetic dyslipidemia and eventually contribute to heart dysfunction [42]. Although the underlying pathogenesis and mechanisms of DCM are complex and multifactorial, here, we summarize some of the mechanisms involved in the glucose overload of DCM in NHPs.

### 3.1. Cardiac Glucose Metabolism Alteration and the Role of Glucose in Cardiac Dysfunction in Diabetic NHPs

Free fatty acids and glucose are two major substrates as the source of energy in the heart and glucose generates 10% to 20% of the energy. Diabetic hyperglycemia seems to be an important cause of the pathogenesis of DCM [33, 43], and reduced glucose oxidation and increased fatty acid oxidation are observed in the diabetic heart [40, 44]. This results in a high consumption of mitochondrial oxygen and contributes to cardiac dysfunction [45, 46]. Insulin plays an important role in maintaining cardiovascular homeostasis and glucose utilization [47]. Insulin resistance is another characteristic of T2D. NHPs with the onset of T2D characterized as impaired pancreatic insulin secretion ability have some abnormal glycemic parameters, including increased fasting glucose level, increased glycated plasma proteins and hemoglobin, delayed/decreased glucose clearance rate, and deteriorated carbohydrate metabolism [26, 48, 49].

One of the limiting steps in cardiac glucose metabolism is glucose uptake by glucose transporters GLUT1 and GLUT4. GLUT1 is responsible for maintaining a basal rate of glucose uptake. GLUT4 is a major mediator of glucose removal from circulation in the adult heart and plays a key role in maintaining whole-body glucose homeostasis. Its translocation to the cell membrane requires insulin, which is impaired in insulin-resistant humans and NHPs [50, 51]. This reduced insulin-stimulated glucose transport is partially compensated by the elevations in plasma glucose-enhanced glucose uptake through mass action [52]. Therefore, although the GLUT4 activity is impaired in the diabetic myocardium, the glycolytic influx is not limited based on enough amount of glucose for hexokinase reaction.

Glycolysis is one of the most important routes of glucose metabolism in cardiomyocytes. Once transported into cardiomyocytes, glucose is broken down to produce pyruvate by glycolysis. In the diabetic heart, the ability of glycolysis and pyruvate oxidation is reduced in different animal models, including NHPs [53–55]. The activity of pyruvate dehydrogenase (PDH), a regulatory enzyme for the balance between cardiac carbohydrate and fat metabolism, is also decreased in T2D, thus, limits pyruvate oxidation. The uncoupling of pyruvate oxidation from glycolysis in diabetic hearts results in accumulated glycolytic intermediates, which further contribute to the development of cardiac dysfunction in diabetes [56, 57].

The previous study has shown that hyperglycemia (glucose overload) can independently regulate diabetic heart glucose metabolism [44]. A study of LV function in NHPs model of dysmetabolism and diabetes has demonstrated that hyperglycemia is strongly associated with the incidence of LV systolic dysfunction (low EF) but insulin treatment has no significant effects in improving the LV systolic dysfunction in T2D NHPs [31]. Another study showed that mechanical dysfunction of cardiomyocytes is correlated with mitochondrial dysfunction in patients with T2D but not with insulin-sensitive obesities. They also demonstrated that mitochondrial dysfunction correlated with HbA1c, not with insulin resistance [58]. This suggests that hyperglycemia, not insulin resistance, is the crucial component of cardiac dysfunction associated with T2D. Moreover, mechanistic and cellular studies have also shown that glucose per se can alter cardiomyocyte and heart contact and contractile properties [59], and that high-glucose exposure induces cardiomyocyte apoptosis [60] and endoplasmic reticulum (ER) stress [61].

Further, there was a relative risk reduction in cardiovascular mortality and hospitalization for HF patients with T2D under the treatment of sodium-glucose cotransporter-2 inhibitors (SGLT2i) [38]. SGLT2i is a class of antidiabetic drugs that reduces glucotoxicity by promoting glycosuria in humans and NHPs [62]. The effect of SGLT2i in cardiac function improvement was also observed in several T2D rodent models [63, 64], thereby supporting the central role of glucose overload in cardiomyopathy development.

### 3.2. High Glucose Increases Oxidative Stress and Mitochondrial Dysfunction in Diabetic NHPs

Excess glucose can increase the production of nicotinamide adenine dinucleotide phosphate hydrogen (NADPH) through the pentose phosphate pathway generated from glucose-6-phosphate (G6P). NADPH is the substrate of cytosolic NADPH oxidase which is known to generate reactive oxygen species (ROS) and served as the principal source of glucose-induced ROS formation in the cells and tissues of diabetic models [65]. Therefore, glucose excess contributes to ROS production and eventually can affect cardiac function. Oxidative stress plays a pivotal role in the pathophysiology of DCM and HF [52, 66]. Mitochondria serve a critical role in energy transduction and intracellular signaling. Interestingly, all the underlying mechanisms of cardiac dysfunction in diabetes are related to mitochondrial injury [67, 68].

Mitochondria disarrangement and impaired mitochondria function were often observed in the diabetic NHP hearts. Mitochondria are the major source of ROS production, and the altered glucose metabolism in diabetic hearts causes mitochondrial oxidative stress evidenced as the excess amount of pyruvate and acetyl-CoA was produced during glycolysis, thus, elevating the production of NADH, an electron carrier. The elevated level of NADH will cause an electron pressure on the mitochondrial electron transport chain, leading to mitochondria dysfunction. The direct role of hyperglycemia in mitochondria dysfunction has been well established in NHPs. A recent study has shown that short-term (1 month) hyperglycemia in cynomolgus monkeys increases mitochondria oxidative stress, and superoxide production, the major form of ROS released from mitochondria, contributes to mitochondria dysfunction [69]. By using cultured NHPs vascular smooth muscle cells with high glucose, Ungvari et al. [70] have shown significantly elevated cellular peroxide levels and mitochondria ROS production.

### 3.3. Accumulation of Advanced Glycation End Products (AGEs) and Activation of Hexosamine Biosynthesis in Diabetic NHPs

Accumulation of advanced glycation end products (AGEs) and increased hexosamine biosynthesis pathway (HBP) has also been found to play major roles in mediating high glucose-induced mitochondria impairment. AGEs, as a group of irreversible adducts from nonenzymatic glucose reactions with proteins (glycation), participate in the pathogenesis of DCM [71]. Previous studies have shown that chronic hyperglycemia alters mitochondrial function through glycation of mitochondria proteins in diabetic rats and mice [72, 73]. Treatment with an AGEs cross-link breaker, ALT-711, significantly improved ventricular function in diabetic rats [74] and reversed the impaired coupling between the vasculature and heart which is common in aging NHPs [75]. Recently, HBP flux has also been involved in the regulation of mitochondrial dysfunction in DCM. O-linked *β*-N-acetylglucosamine moiety (O-GlcNAc), supplied by HBP flux, is O-linked on the serine and threonine residues of numerous proteins by O-GlcNAc transferase (OGT). Previous studies have shown that the O-GlcNAcylation process is highly activated in diabetic hyperglycemia in rodents [76] and stressed NHPs [77]. Hu et al. have found that increased O-GlcNAcylation of mitochondrial proteins impairs mitochondrial function in cardiomyocytes exposed to high glucose [78]. A recent study has demonstrated that dysregulation of O-GlcNAcylation in diabetic cardiac mitochondria plays a key role in mitochondrial dysfunction [79]. Taken together, these results highlight the important role of cardiac O-GlcNAcylation in regulating mitochondrial function in the diabetic heart.

### 3.4. High Glucose Affects the Response to Cardiac Resynchronization Therapy with Defibrillator (CRT-d) in Diabetic NHPs

Cardiac resynchronization therapy with defibrillator (CRT-d) was often used in HF patients to improve cardiac contractile function, clinical outcomes, and quality of life and to reverse ventricular remodeling [80]. CRT-d was also found to be beneficial for the HF NHPs with the fibrotic myocardial disease based on clinical signs [81]. However, as reported previously, high glucose and T2D are related to an increased percentage of cardiovascular disease (CVD) progression towards HF, which is closely correlated to the proinflammatory and prothrombotic state. Several studies reported that insulin-dependent T2D patients showed a worse prognosis after CRT-d. The researcher believed these were attributed to the alterations in the mitogenic and metabolic pathways in those patients, leading to a poor CVD pathogenesis progression [22]. Recently, authors showed that T2D and other risk factors (hypertension, overweight) influenced the CRT-d's functionality in HFrEF patients by a proarrhythmic status, then can result in a reduced survival rate and higher hospital admissions rate in these patients [80, 82].

Recent findings demonstrated that the ameliorative effects of CRT-d treatment such as improved myocardial ventricular geometry and functional capacity in humans with T2D are determinants of reduced hospitalizations and arrhythmias [83]. Moreover, Sardu et al. found that the multipolar CRT-d pacing was much better than the bipolar CRT-d pacing with a significant reduction of hospitalizations for HF worsening, PNS (phrenic nerve stimulation), LV catheter dislodgment, and atrial fibrillation events, and multipolar CRT-d pacing could be an independent predictor of above events [84]. Furthermore, a recent new technique, an automatic-optimized CRT-d with SensoR technology was found to be superior to the echo-guided CRT-d with respect to the significant increase of CRT-d responder rate and the significant reduction of hospital admissions for HF worsening and cardiac deaths. Therefore, this could be used to reduce the worse prognosis in HF patients with T2D [85]. However, the loss of CRT-d effects was often observed in patients with T2D, which may be due to multiple molecular, electrical, and metabolic cardiac changes. In this setting, the new incretin drugs such as glucagon-like peptide 1 receptor agonists (GLP-1 RA) have been used in HF patients with T2D, and interestingly, the risk of hospitalization for those patients was not increased. Therefore, GLP-1 RA in addition to conventional hypoglycemic therapy was also introduced to HF patients with T2D to improve the CRT-d responder rate. It was confirmed that GLP-1 RA therapy in addition to standard hypoglycemic drugs significantly reduced inflammation, B type natriuretic peptide values (linked to the improvement of LVEF), hemodynamic effects, arrhythmic burden, and hospitalization for HF worsening in failing heart patients with T2D treated by CRT-d [86]. Thus, this describes the glycemic control necessary to successfully manage these HF patients in conjunction with CRT-d therapy.

### 3.5. Experimental Diabetic NHP Models

#### 3.5.1. Spontaneous Diabetic NHP Models

Several NHP species develop obesity and diabetes spontaneously, and these NHPs exhibit clinical characteristics of obesity and diabetes including insulin resistance, hyperinsulinemia, dyslipidemia, progressive hyperglycemia, and pancreas pathology are similar to humans [87]. This makes these NHPs valuable resources for studying therapeutic interventions and molecular and cellular mechanisms of human T2D as well as related cardiovascular disease (CVD). Macaques, including cynomolgus macaques and rhesus macaques, are the most widely studied spontaneous diabetic NHP models [88].

Previous studies have shown that approximately 30% of middle-aged cynomolgus monkeys (>15 years-old) exhibit impaired glucose tolerance (IGT) with moderate hyperinsulinemia before becoming overt hyperglycemia [48]. Like humans, cynomolgus monkeys progress from IGT to T2DM with a decrease in pancreatic insulin secretion. T2D monkeys always exhibit increased glycated hemoglobin (HbA1c), hyperglycaemic, dyslipidemic with severe insulin resistance, and decreased glucose clearance rate for years before clinical intervention is needed [89]. A recent study in spontaneous obese, dysmetabolic, and diabetic cynomolgus monkeys has demonstrated LV systolic and diastolic dysfunctions in these NHPs, similarly to that in diabetic patients [31].

The increased baseline of insulin secretion and impaired insulin response to glucose challenge are the earliest characteristics in T2D rhesus monkeys. Wang et al. previously screened 3 middle and elderly aged rhesus monkeys from 100 rhesus monkeys by glucose tolerance test and glycosuria test [90]. This indicated the morbidity rate of spontaneous diabetes in rhesus monkeys is consistent with that observed in humans. Rhesus monkeys also exhibit age-related clinical diabetes features including decreased insulin sensitivity and decreased insulin response to glucose challenge [91, 92]. By echocardiography and magnetic resonance imaging, 6 out of 15 T2D rhesus monkeys were diagnosed with diastolic dysfunction [93]. Importantly, female NHPs have menstrual cycles that closely approximate that of humans and gestational diabetes (GD) has been demonstrated in cynomolgus [94] and rhesus monkeys [95]. GD monkeys exhibit elevated glucose and insulin and deliver macrosomic infants, similar to women with GD [94], and there is a risk of developing T2D following GD in monkeys as in humans [48].

#### 3.5.2. Inducing Diabetic NHP Models

Given that the disease progression is relatively slow and the percentage of NHPs progressing to overt diabetes is small, researchers successfully induced T2D monkey models by diet regimens. These T2D monkey models are more closely resemble human T2D pathogenesis as compared with models induced by streptozotocin, a specific *β*-cell toxin. Streptozotocin-induced diabetic monkeys, presenting different extent of islet damage, are usually not insulin resistant but require exogenous insulin treatment [96].

Many NHPs fed with high fat and/or high sugar diet progressively developed from insulin resistance and impaired glucose tolerance to T2D, as in humans [97]. Bremer et al. demonstrated that a high fructose dietary additive was able to induce metabolic syndrome features including central obesity and T2D in rhesus monkeys [98]. Kavanagh et al. have shown that long-term administration of trans fatty acids in monkeys leads to significant weight gain and insulin resistance in this NHP model [99]. Taken together, the above findings also provided data on further optimizing dietary strategies to establish more effective animal models for studying human T2D.

As discussed above, glucose overload plays a central role in the development of DCM, therefore, glucose control is the primary objective for the treatment of DCM. Caloric restriction is a successful intervention for the management of T2- related metabolic abnormalities both for humans and NHPs [99, 100]. A caloric restriction study in cynomolgus and rhesus monkeys successfully decreased plasma glucose and insulin level and increased insulin sensitivity [101, 102]. Other treatments including SGLT2i, which is a new class of antidiabetic drugs that reduces glucotoxicity by promoting glycosuria, have been conducted in NHPs [62] and used to treat HF patients with T2D [38].

Metformin and thiazolidinedione are used to improve insulin resistance and glucose uptake. Sulfonylureas, glucagon-like peptide-1 (GLP-1) agonists, and dipeptidyl peptidase-4 (DPP-4) inhibitors are also used in both humans and NHPs to stimulate insulin secretion to lower blood glucose [103].

## 4. Advantages and Limitations of NHP Models

The most important advantages of NHP models are the genetic and physiological similarities to humans, and the genomes of some NHP species have been sequenced [104, 105]. The second advantage in NHP studies is the complete control of diet, drug treatment regimens, which are quite difficult to be controlled in human subjects. Additionally, the biopsies of different organs can be sequentially acquired through minimally invasive approaches in the studies of NHPs, which is not possible in humans. Nevertheless, there are very few studies using nonhuman primate aging models likely because of their limited existing numbers, high cost, and potential ethical concerns. There are also very limited facilities that can provide support for NHP research due to the challenges of limited resources and required technical expertise.

## 5. Conclusions of the Importance of NHPs in DCM Research

**Figure 1:**
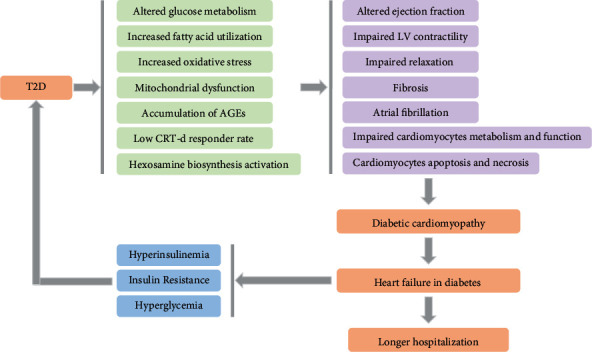
The effects of type 2 diabetes (T2D) in patients with heart failure (HF). Patients with T2D can lead to altered glucose metabolism, increased fatty acid utilization and oxidative stress, mitochondrial dysfunction, accumulation of advanced glycation end products (AGEs), lower cardiac resynchronization therapy with defibrillator (CRT-d) responder rate, and hexosamine biosynthesis activation. These effects cause altered ejection fraction, impaired left ventricular (LV) contractility, impaired relaxation, heart fibrosis, atrial fibrillation, impaired cardiomyocytes metabolism and function, cardiomyocytes apoptosis, and necrosis, all of which contribute to diabetic cardiomyopathy, leading to increased heart failure in diabetes. T2D patients with HF present longer hospitalization and a higher rate of hyperinsulinemia, insulin resistance, and hyperglycemia, which can further exacerbate T2D progression.

Given the fact that T2D is a major risk factor for HF and hyperglycemia is strongly associated with diastolic dysfunction in diabetes patients, summarized in Figure 1, and the investment in T2D and HF research by the whole society, reliable preclinical models of human disease are urgently needed. NHPs are such an invaluable translational model in the study of human DCM pathology. First, NHPs show the greatest similarities to the disease of humans. Second, the genomes of the commonly used NHPs in biomedical research have been sequenced. Third, NHP models served as unique models for establishing the safety and efficacy of novel drug development in humans.

## Data Availability

The data supporting this review are from previously reported studies and datasets, which have been cited.
